# Does Sympathy Motivate Prosocial Behaviour in Great Apes?

**DOI:** 10.1371/journal.pone.0084299

**Published:** 2014-01-08

**Authors:** Katja Liebal, Amrisha Vaish, Daniel Haun, Michael Tomasello

**Affiliations:** 1 Excellence Cluster “Languages of Emotion”, Department of Education and Psychology, Evolutionary Psychology, Freie Universität Berlin, Berlin, Germany; 2 Department of Psychology, University of Portsmouth, Portsmouth, United Kingdom; 3 Department of Developmental and Comparative Psychology, Max Planck Institute for Evolutionary Anthropology, Leipzig, Germany; Institut de Biologia Evolutiva - Universitat Pompeu Fabra, Spain

## Abstract

Prosocial behaviours such as helping, comforting, or sharing are central to human social life. Because they emerge early in ontogeny, it has been proposed that humans are prosocial by nature and that from early on empathy and sympathy motivate such behaviours. The emerging question is whether humans share these abilities to feel with and for someone with our closest relatives, the great apes. Although several studies demonstrated that great apes help others, little is known about their underlying motivations. This study addresses this issue and investigates whether four species of great apes (*Pongo pygmaeus, Gorilla gorilla, Pan troglodytes, Pan paniscus*) help a conspecific more after observing the conspecific being harmed (a human experimenter steals the conspecific’s food) compared to a condition where no harming occurred. Results showed that in regard to the occurrence of prosocial behaviours, only orangutans, but not the African great apes, help others when help is needed, contrasting prior findings on chimpanzees. However, with the exception of one population of orangutans that helped significantly more after a conspecific was harmed than when no harm occurred, prosocial behaviour in great apes was not motivated by concern for others.

## Introduction

Prosocial behaviours such as helping, comforting, or the sharing of resources or information are central to human social life [1], [2] and emerge early in ontogeny [3]. For example, infants as young as 12 months inform an adult who is searching for an object by directing them with a pointing gesture [4]. Around 14 – 18 months they help obtaining objects that are out of an adult’s reach or remove obstacles that prevent an adult from completing an action [5], even without encouragement or praise [6], [7]. It has thus been suggested that children are altruistic by nature, with their initial altruistic tendencies being further developed and influenced by their subsequent social interactions with others [8], [9]. Thus, children’s altruistic behaviour increases with age, regardless of their socio-economic environment or cultural background [10], [11], even when facing adversity such as natural disasters [12].

The question thus arises, what motivates children to help others? According to Hoffman [13], empathy and sympathy are the major motivations for prosocial behaviours. While empathy represents the ability to feel *with* someone, sympathy refers to the ability to feel *for* someone and to experience concern for others, often leading to prosocial behaviours to ease the other’s distress [14]. Both empathy and sympathy are considered essential skills to maintain and regulate the complex social life of humans. For example, individuals who are empathic or sympathetic are more likely to act in prosocial ways and thus less likely to show antisocial behaviours such as aggression [15], [16].

A variety of studies showed that from an early age, children show empathic and sympathetic responses when observing others’ distress (for a review, see [17]). For example, soon after birth, infants cry in response to others’ crying or distress, which indicates at least some precursor to empathy such as emotional contagion [18]. Between the first and second years of life, children start to perform prosocial actions in response to others’ distress, with these actions increasing in frequency and variety over development [19]. A recent study demonstrated that even in the absence of any cues indicating another person’s distress, children as young as 18 months sympathize with an adult who has been harmed and subsequently act prosocially towards the adult such as by sharing resources with that person ([20], using a procedure adapted from [21]). In that study, children in one condition witnessed an adult being harmed (e.g., her necklace was taken by another person) and children in a second condition witnessed the adult not being harmed (her necklace was not taken). Children’s concerned looks towards the adult were assessed while the adult was being harmed or not harmed, and children’s subsequent prosocial behaviour towards the adult who was harmed or not harmed was also assessed. The study revealed that children showed greater concern if the adult was harmed than if the adult was not harmed, and also showed more prosocial behaviour towards the adult if that person had previously been harmed than if the adult had not been harmed. Moreover, the degree of children’s concern for the adult correlated positively with the degree of their subsequent prosocial behaviour towards that person, suggesting that concern for the victim motivated their prosocial behaviour. Together these studies suggest that humans are prosocial by nature and that their ability to empathize and sympathize with others is a major factor motivating such prosocial behaviours [8], [9], [13].

The nature of prosocial behaviour in species other than humans is still debated. Some argue that humans are the only species that show altruistic behaviours [22], while others suggest that it is unlikely that this trait only emerged in humans [23]. To address this question, several experimental studies have investigated prosocial behaviours in various species of nonhuman primates, including New World monkeys, Old World monkeys, and great apes ([24]–[27], for recent reviews see [28], [29]). Because of their close relatedness with humans, much research has focused on chimpanzees; however, this research has yielded inconsistent conclusions. For example, there is little evidence that chimpanzees distribute food to other conspecifics, even if it is at no or little costs to themselves [30]–[32], and even in dyads of closely related individuals such as mothers with their offspring [33].

More positive results are reported from studies that centre to a lesser extent on food and that look at other behaviours such as helping to obtain an object or providing a tool to get out-of-reach food. Several studies by Warneken and colleagues [5], [6], [34] focused on situations that require instrumental or targeted helping, which is defined as help based on the cognitive appreciation of the situation or needs of others [23]. For example, chimpanzees helped to obtain objects that were out of a human’s reach, but as opposed to 18-month-old children, they did not help in more complex tasks that involved the removal of physical obstacles, or helping by correcting wrong results or wrong means [5]. In both chimpanzees and children, the presence of a potential reward did not increase the probability of helping. Further research clarified that helping in chimpanzees was neither limited to familiar situations nor to interactions with humans [6]. However, Yamamoto and colleagues demonstrated that chimpanzees rarely spontaneously offered a tool to a conspecific in need, since those transfers were more likely to occur if the potential recipient performed different kinds of gestures or vocalizations [35]. In summary, there is some, though inconsistent evidence that chimpanzees and other nonhuman primates show prosocial behaviours, but this seems to be limited to some rather specific situations or contexts.

What motivates prosocial behaviours in nonhuman primates remains an open question. While some argue based on comprehensive observations in both captive and wild settings that the abilities to empathize with and to feel concern for others are major motivations for prosocial behaviours in nonhuman primates [14], [23], [36], others suggest that it is important to consider alternative explanations for such apparently prosocial activities [37]. For example, Gilby [38] concluded that meat-sharing in chimpanzees is most likely guided by the motivation to avoid harassment by other group members. Thus, the individual that possesses the food offers a share to others to stop their requesting behaviours, indicating a much more self-oriented motivation [39].

Taken together, the increasing body of research on prosocial behaviour, especially in chimpanzees, offers little explanations in regard to the underlying motivations. The current study was thus aimed at investigating whether the prosocial behaviour of four species of great apes is motivated by sympathy for others. Following the procedure of Vaish et al. [20], we investigated whether great apes show greater prosocial behaviour towards a conspecific who has been harmed than towards a conspecific who has not been harmed, under the assumption that greater prosocial behaviour towards a conspecific who has been harmed would suggest that sympathy drives prosocial behaviour in apes as it does in humans. In order to avoid any direct competition over food, prosocial behaviour was assessed in the form of helping. More specifically, we measured whether great apes transfer a tool to another conspecific so that this individual can obtain food that is out of reach. If great apes are sensitive to the affective states of others and thus feel concern for them, they should transfer more tools in the condition in which the other individual was previously harmed compared to the condition in which no harm occurred.

## Methods

### Ethics statement

Research was conducted at the Wolfgang Köhler Primate Research Center (WKPRC) at Zoo Leipzig, Germany, the Orangutan Care Center and Quarantine, Pasir Panjang (OCCQ), Kalimantan Tengah, Indonesia, and the Ngamba Island Chimpanzee Sanctuary, Lake Victoria, Uganda. All procedures were non-invasive and research complied with the recommendations of the Weatherall report, the EAZA Code of Practice Article 4: Research, and the WAZA Ethical Guidelines for the Conduct of Research on Animals by Zoos and Aquariums. The apes voluntarily participated in the study, could choose to stop participating at any time and were never food or water deprived. Rewards were highly valued food-items. All apes at the WKPRC and to a lesser extend at OCCQ and Ngamba Island had previously participated in various studies on social and physical cognition. The research was ethically approved by an internal ethics committee at the Max Planck Institute for Evolutionary Anthropology consisting of scientists (Prof. M. Tomasello, Dr. J. Call, Dr. D. Hanus), zoo keepers (head keeper F. Schellhardt, assistant head keeper M. Lohse), and a veterinarian (Dr. A. Bernhard) as well as the Chimpanzee Sanctuary & Wildlife Conservation Trust. The research strictly adhered to the legal requirements of the involved countries and was approved by the Ugandan Wildlife Authorities and the Ugandan National Council for Science and Technology (Uganda) as well as the Ministry of Research and Technology (Indonesia). At WKPRC, the different species of great apes are housed in groups in semi-natural indoor (175 – 246 m^2^) and outdoor enclosures (1400 – 2300 m^2^) containing climbing structures such as trees, ropes and platforms as well as a variety of enrichment devices, and spend the night in a series of interconnected sleeping rooms (32 – 40 m^2^). At Ngamba Island, chimpanzees are allowed to roam freely on the 40 ha island covered with tropical rain forest during the day and spend the night in seven interconnected sleeping rooms (approx. 140 m^2^) (Ngamba Island). At OCCQ, apes live in peer groups in enclosures with excursions to the forest every other day, with the exception of one partly paralyzed individual (Bali). Research was conducted in the sleeping and/or observation rooms. All apes have regular feeding schedules and water ad lib. With the exception of the chimpanzees at Ngamba Island who spend most of their day in the forest, apes at WKPRC and OCCQ receive different enrichment activities. At WKPRC, this includes shaking boxes and poking bins permanently installed in their enclosures, as well as the daily provision with different types of enrichment material at 3.30 pm with at least one item per individual (e.g., jute and paper parcels filled with seeds; for more information, see http://wkprc.eva.mpg.de/english/files/enrichment.htm). At OCCQ, orangutans receive different types of enrichment once per day, including towels, ice cubes, coconuts, and balls filled with seeds.

### Subjects

Four species of great apes (orangutans, gorillas, chimpanzees and bonobos) were tested at the WKPRC at Zoo Leipzig. Furthermore, two populations housed at sanctuaries were tested, including orangutans at the OCCQ Pasir Panjang, and chimpanzees at Ngamba Island (supporting information, Table S1). In total, 21 orangutans (seven Sumatran orangutans (*Pongo pygmaeus abelii,* one male, six females*; M_age_ = *18.5 years, *SD = *11) at WKPRC at Zoo Leipzig, 14 Bornean orangutans (*Pongo pygmaeus pygmaeus,* eight females, six males*; M_age_ = *8.5 years, *SD = *1.3) at OCCQ Pasir Panjang), four gorillas (*Gorilla gorilla*, one male, three females*; M_age_ = *13.5 years, *SD = *11.4), 29 chimpanzees (*Pan troglodytes*, six at WKPRC: one male, five females; *M_age_ = *12.8 years, *SD = *3.5; 23 at Ngamba Island: eleven males, twelve females; *M_age_ = *15.7 years, *SD = *5), and six bonobos (*Pan paniscus,* three males, three females; *M_age_ = *14.3 years, *SD = *7.5) participated in this study. In total, this study included 23 males and 37 females with their ages ranging from 4 to 36 years (*M = *13.8, *SD = *6.78). The majority of the great apes at the WKPRC in Leipzig were mother-reared, while a larger proportion of individuals in both sanctuaries had more and closer contact with humans in early stages of their life.

### Experimental setting

Only those individuals were included in this study that successfully passed a pre-test in which they needed to use a stick to rake in a minimum of six food items in two consecutive sessions. The purpose of this pre-test was to check whether they were capable of using a stick and to familiarize them with the testing procedure where they also had to use a stick to obtain food that was out of their reach.

The apes were tested in pairs (supporting information, Table S2). Each individual that successfully passed the pre-test was randomly assigned to a role as victim (interacting with the human experimenter, E) and/or as helper (interacting with the victim) (supporting information, Table S1). Thus, some individuals were victims *and* helpers, but they never participated in each of these roles in more than three dyads. Within a dyad, victims and helper never switched roles. This resulted in 29 orangutan dyads (11 at OCCQ, 18 at WKPRC), five gorilla dyads, 27 chimpanzee dyads (15 at the WKPRC, 12 at Ngamba Island), and 12 bonobo dyads.

### Procedure

The great apes were tested in two adjacent rooms that were separated by mesh. The victim sat opposite to E, while the helper was in the neighbouring room. One camera recorded the interactions between the victim and E, the second camera recorded the interactions between the victim and the helper, and third third camera focussed on the helper. Because of the spatial arrangement of the two rooms, however, it was often not possible to record the behaviour of the helper.

First, each of the two individuals of a dyad participated in a warm-up trial separately. The warm-up trial was identical to the pre-test to ensure that they were familiar with the setting and knew that it was essential to use a stick to obtain the food items out of their reach. Then this dyad participated in four different conditions. There were two experimental conditions (Take and Give) and two control conditions (Control 1: No food, and Control 2: No victim). Each dyad was tested only once in each of the four conditions, with each condition being presented on a different day. The order of conditions was randomized across dyads.

The Take and Give conditions both began with an observation phase, in which the helper witnessed the interactions between the victim and the human. In the Take condition, E sat opposite to the victim and checked that the helper was attending. Then E took a grape and pretended to hand it over to the victim, before pulling her hand back and eating the grape herself. This was repeated until 30 s elapsed and E ate an average of 3.4 grapes (*SD = *0.50). Then E remained in front of the victim and looked at the victim for another 30 s without any further interactions. In the Give condition, E took a grape and moved it towards the victim, but now handed it over to the victim. This was repeated until 30 s elapsed and E gave on overage 3.5 grapes (*SD = *0.64). Then E remained in front of the victim and looked at the victim for another 30 s without any further interactions. In both conditions, the observation phase was followed by a prosocial phase, during which E placed six banana slices on the table out of the victim’s reach and gave three sticks to the helper in the adjacent room, before leaving the testing area for 3 minutes.

The two control conditions were conducted to control for the fact that the apes might simply transfer sticks from one room to the other without considering whether any help is needed. In contrast to the Take and Give conditions, they only consisted of a prosocial phase, with the modification that in Control 1: No food, no food was placed on the table in front of the victim, while in Control 2: No victim, no victim was present that needed the sticks to obtain the food.

### Coding

The coding of the video footage included the identification of stick transfers as well as arousal and requesting behaviours of the victim.

**Stick transfer:** A stick transfer from the helper to the victim was coded as present if any of the helper’s sticks were identified in the victim’s room, or as absent if the helper still had all three sticks at the end of the trial. Furthermore, if a stick transfer was coded as present, it was classified as an *Offer* (with the helper offering at least one stick to the victim, either after or without a preceding request from the victim), *Passive* (if the victim could reach a stick and took it without the helper's resistance), or *Unclear* (if the stick transfer was not visible on the video or the kind of transfer could not be determined).

**Behaviours:** Two different types of the victim’s behaviours were coded in the Take and Give conditions only. First, any behaviour that indicated the arousal of the victim (hand shake, head shake, muzzle wipe, rattling of the mesh, scratching, spitting, vocalization) were coded. For this measure, we coded the occurrence of these behaviours during the observation phase of the Take and Give conditions (60 s), and during the prosocial phase of the Take and Give conditions (180 s). Second, we coded any requesting behaviours (putting the fingers through the mesh, extending the arm with the palm up) that the victim directed towards the helper during the prosocial phase of the Take and Give condition.

**Reliability:** To ensure reliability for the occurrence of stick transfers and behaviours (arousal behaviours and requests of the victim), a person unfamiliar with the purpose of this study coded 20% of the data. Cohen’s kappa was used to measure the degree of concordance. For the occurrence of stick transfers, Kappa was 0.73 (93.3 % agreement), which corresponds to a good level of agreement [40]. For the occurrence of behaviours, Kappa was 0.86 (89.0 % agreement), which corresponds to a very good level of agreement [40].

### Statistics

Data analysis was conducted in two parts. The first part consisted of analysing stick transfers across the different conditions to see whether great apes transferred more sticks when help was actually needed and whether they transferred sticks differentially when the victim was harmed compared to when the victim was not harmed. The second part consisted of analysing the victim’s behaviours towards E and the helper, including any behaviour that indicated the arousal of the victim as well as request behaviours, to see whether the victim’s behaviour influenced the likelihood of a stick transfer.

Statistics were calculated using R 2.14.2 [41], namely the function lmer of the R-package lme4 [42]. To consider the identities of the two interacting individuals and thus a possible influence on the individuals tested, Generalized Linear Mixed Models (GLMM) [43] were conducted. For each model, the statistical significance of the full model was tested by comparing it to a null model (with the fixed effects excluded) by using a likelihood ratio test (R function “anova”) [44]. To analyse whether the frequency of stick transfers differed between conditions, the full model with the fixed effects *species*, *conditions* and the interaction between them and *victim* and *helper* as random effects was compared to the null model only including the random effects. To analyse whether the frequency of stick transfers differed between populations (zoo or sanctuary) within a species, the full model with the fixed effects *species, population* and *condition* and all interactions between them up to third order and the random effects *victim* and *helper* was compared to a null model with the factor *population* and all interactions with it excluded. To analyse whether the victim’s arousal behaviour in the observation phase differed between the Take and Give conditions, the full model with the fixed effects *species* and *condition* and the random effects *victim* and *helper* was compared to the null model only comprising the random factors. The victim’s arousal in the prosocial phases of the Take and Give conditions was analysed in the same way. Models with stick transfer as the response were fitted with binomial error structure and logit link function, and models with arousal behaviour as a response were fitted assuming poisson error structure and log link function. For the poisson models there was no indication that overdispersion was an issue (arousal during observation phase: χ^2^ (137) * = *114.2, *p = *0.923, dispersion parameter* = *0.833; arousal during prosocial phase: χ^2^ (137) * = *125.58, *p = *0.748, dispersion parameter* = *0.917).

## Results

### Stick transfers

**Instances and types of stick transfers:** Overall, stick transfers occurred in only 11 % (N* = *32) of all trials. Almost half (N* = *15) of all stick transfers were observed in the Take condition and about one third (N* = *11) occurred in the Give condition (for examples of stick transfers in orangutans (Video S1 and Video S2) and gorillas (Video S3), see supporting information). In the two control conditions together, there were six instances of stick transfers. Table 1 shows the proportions of trials with stick transfers in the four conditions for each of the four species. Since only one stick transfer was observed among the gorillas and bonobos, respectively, both species were excluded from the following analyses.

**Table 1:** Proportion of trials with stick transfers (with frequencies in brackets) in each condition.

Species (# of dyads in brackets)	Take	Give	Control 1: No food	Control 2: No victim
**Orangutans** (29):	41.4 (12)	27.6 (8)	10.4 (3)	0
Orangutans Leipzig (18)	44.4 (8)	44.4 (8)	10.4 (3)	0
Orangutans Pasir Panjang (11)	36.4 (4)	0	0	0
**Gorillas** (5)	20 (1)	0	0	0
**Chimpanzees** (27):	3.7 (1)	11.1 (3)	7.4 (2)	3.7 (1)
Chimpanzees Leipzig (15)	0	0	6.7 (1)	0
Chimpanzees Ngamba Island (12)	8.3 (1)	25 (3)	8.3 (1)	8.3 (1)
**Bonobos** (12)	8.3 (1)	0	0	0

With regard to types of stick transfers, of the 23 stick transfers among the orangutans, almost three quarters (N* = *17) were offers and only one was a passive transfer. In contrast, more than half of the seven stick transfers in chimpanzees were passive transfers (N* = *4) and only in two instances were sticks offered to the victim. The remaining stick transfers (orangutans: 5; chimpanzees: 1) were classified as unclear.

**Figure 1:**
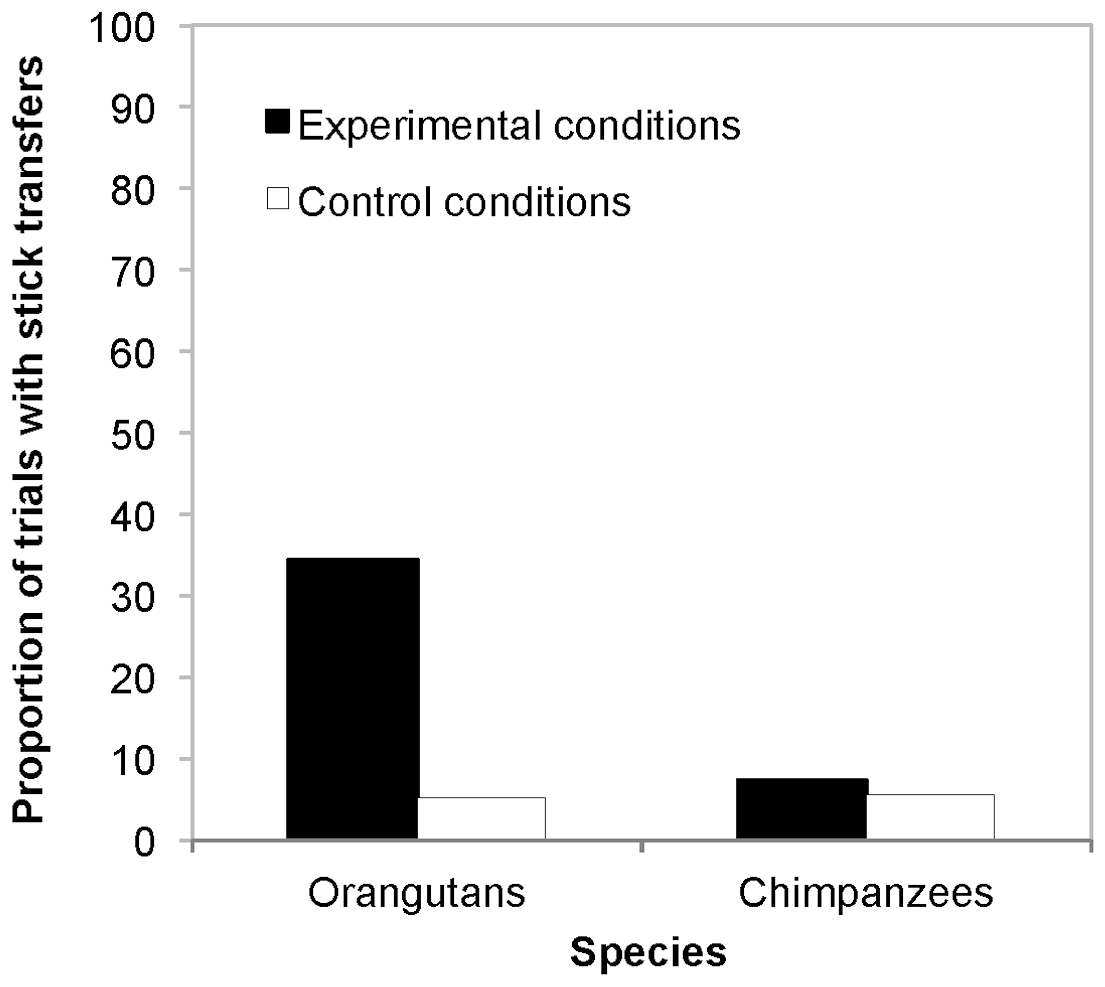
Proportion of trials with stick transfers. Orangutans transferred more sticks in the Experimental conditions (Take and Give) than in the Control conditions (Control 1: No food and Control 2: No victim), while the occurrence of stick transfers in chimpanzees did not differ between the Experimental and Control conditions.

**Do chimpanzees and orangutans give sticks when they are needed?** Here we analysed whether overall, chimpanzees and orangutans helped when a stick was needed, regardless of whether any harming occurred or not. Thus, while in both the Take condition and the Give condition a stick was essential to obtain the food, no stick was needed in the two control conditions. Therefore, for this analysis, we combined the stick transfers in the Take and Give condition (experimental conditions) and compared them to the combined stick transfers in the Control 1: No food and Control 2: No victim condition (control conditions). The comparison of the full against the null model was clearly significant, indicating an effect of the factors species and condition or their interaction on the occurrence of stick transfers (χ^2^ (3) * = *24.64, *p*<0.0001). The comparison of stick transfers in the experimental and control conditions revealed a significant interaction between species and condition (*χ^2^* (1) * = *4.83, *p = *0.028). Post-hoc tests found a significant difference between the two conditions for orangutans (χ^2^ (1) * = *20.07, *p*<0.0001), such that orangutans transferred more sticks across the experimental conditions (34.5 %) than across the control conditions (5.2 %). However, this difference did not emerge for chimpanzees (experimental conditions: 7.4 %, control conditions: 5.56 %) (χ^2^ (1) * = *0.20, *p = *0.653). Thus, while orangutans gave sticks selectively more when the other individual needed them, chimpanzees' helping behaviour did not differ between the experimental and control conditions (Figure 1).

**Do chimpanzees and orangutans transfer more sticks after the victim was harmed?** We conducted this analysis to investigate whether chimpanzees and orangutans differentiate between conditions in which another conspecific was harmed compared to when no harm occurred and thus would transfer more sticks in the Take compared to the Give condition. The comparison of the full against the null model was clearly significant, indicating an effect of the factors species (orangutans, chimpanzees) and condition (Take, Give, Control 1: No food, Control 2: No victim) on the occurrence of stick transfers (χ^2^ (7) * = *38.28, *p*<0.0001). Considering stick transfers in the Take and Give condition only, there was a significant interaction of condition and species (χ^2^ (3) * = *9.37, *p = *0.025). Post-hoc tests found that orangutans did not transfer sticks differentially across the Take and Give conditions (χ^2^ (1) * = *1.99, *p = *0.159), while chimpanzees transferred more sticks in the Give than in the Take condition (χ^2^ (1) * = *10.11, *p = *0.001). These results show that neither orangutans nor chimpanzees helped more in the Take condition, i.e., after they witnessed a conspecific being harmed.

**Do frequencies of stick transfers differ between populations of one species?** For both orangutans and chimpanzees, two different kinds of captive populations were tested, one of which was housed at a zoo and one at a sanctuary. The comparison of the full model with the fixed effects species (orangutans, chimpanzees), condition (Take, Give, Control 1: No food, Control 2: No victim), and population (zoo, sanctuary) and all interactions between them up to the third order, and victim and helper as random effects was compared to the null model with the factor population and all interactions with it excluded almost reached significance (χ^2^ (8) * = *15.31, *p = *0.053). This suggests an effect of the factor population on the occurrence of stick transfers, but the three-way interaction of the factors population, species, and condition was not significant (χ^2^ (3) * = *0, *p = *1). However, the test of the two-way interactions revealed a significant interaction between population and species (χ^2^ (1) * = *12.06, *p = *0.0005) and between population and condition (χ^2^ (3) * = *9.81, *p = *0.02). When species were analysed separately in regard to the stick transfers in the Take and Give condition, no interaction between condition and population was found for chimpanzees (χ^2^ (1) * = *0, *p = *1), but there was an interaction for orangutans (χ^2^ (1) * = *6.06, *p = *0.014). Thus, in contrast to orangutans in zoos that did not differentiate between Take and Give condition (χ^2^ (1) * = *0, *p = *1), orangutans in the sanctuary gave significantly more sticks in the Take than in the Give condition (χ^2^ (1) * = *6.44, *p = *0.011).

### Analysis of behaviours

Unlike the previous analyses, gorillas and bonobos were also included in the following analyses to see whether their communicative behaviour offers possible explanations for the lack of stick transfers in those two species. Two sets of analyses were conducted, which considered the mean frequencies of communicative behaviours in the different conditions.

**Does the victims’ arousal differ between conditions?** This set of analyses concerned the victims’ behaviours that would indicate any arousal during the observation phase of the Take or Give condition while interacting with E or during the prosocial phase that followed the Take and Give conditions. If victims were more aroused in the Take compared to the Give condition, this would indicate that E’s stealing of food in the Take condition had an effect on the victim’s behaviour, which in turn could trigger prosocial actions by the helper and thus the transfer of sticks.

**Figure 2:**
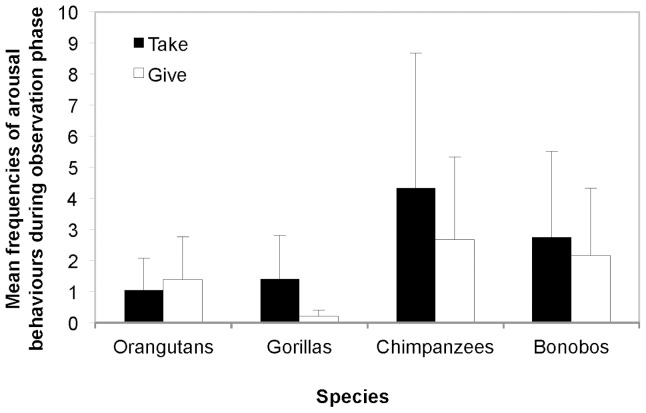
Mean frequencies of arousal behaviours of the victim during the observation phase. There was a significant interaction between condition and species (*p = *0.011), and a main effect of condition (*p = *0.08) and of species (*p = *0.015). African great apes, but not orangutans showed more arousal behaviour when interacting with the experimenter in the observation phase of the Take compared to the Give condition (each lasting 60 seconds). Error bars indicate SD.

First, when considering the mean frequencies of the victim’s arousal behaviours in the observation phases of the Take and Give condition, the comparison of the full against the null model was clearly significant (χ^2^ (7) * = *28.55, *p*<0.001). The interaction between condition and species was significant (χ^2^ (3) * = *11.05, *p = *0.011), and there was a main effect of condition (χ^2^ (1) * = *7.09, *p = *0.008) and of species (χ^2^ (3) * = *10.40, *p = *0.015). These results show that when interacting with E, victims displayed higher arousal in the Take compared to the Give condition in all African apes, but not orangutans (Figure 2).

**Figure 3:**
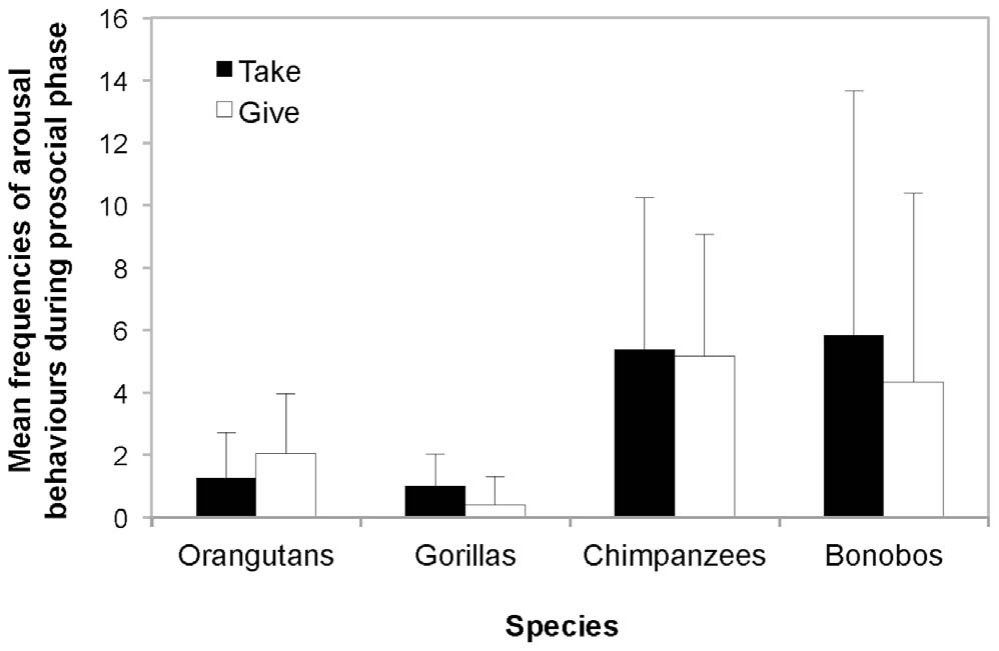
Mean frequencies of arousal behaviours of the victim during the prosocial phase. There was a significant interaction between condition and species (*p = *0.021) and a main effect of species (*p = *0.001), but no main effect of condition (*p = *0.859). Bonobos and chimpanzees showed more arousal behaviours in the prosocial phases of the Take and Give condition (each lasting 180 seconds) than orangutans and gorillas. However, only gorillas and bonobos displayed marginally more arousal in the prosocial phase of the Take condition compared to the Give condition. Error bars indicate SD.

Second, in regard to the mean frequencies of the victim’s arousal behaviours in the prosocial phases of the Take and Give condition, the comparison of the full against the null model was significant (χ^2^ (7) * = *27.14, *p*<0.001). There was a significant interaction between condition and species (χ^2^ (3) * = *9.71, *p = *0.021) and a main effect of species (χ^2^ (3) * = *17.40, *p = *0.001), but no main effect of condition (χ^2^ (1) * = *0.03, *p = *0.859). While gorillas and bonobos displayed marginally more arousal in the prosocial phase of the Take condition, orangutans showed the opposite trend (Figure 3).

**Does the frequency of the victim’s requests correlate with stick transfers?** This set of analyses concerned the requesting behaviour of the victims in the prosocial phases of the Take and Give conditions. We analysed whether the helper was more likely to give a stick if the victim performed requesting behaviours, and if species differed in regard to their mean frequencies of these requests. A three-way interaction between species, condition, and requests towards the helper that controlled for an influence of requests was not significant (χ^2^ (1) * = *1.26, *p = *0.26). This indicates that the requesting behaviour of the victim towards the helper had no effect on the frequency of stick transfer. Furthermore, species did not differ in regard to their frequencies of requesting behaviours towards the helper (χ^2^ (3) * = *6.09, *p = *0.107), demonstrating that the higher frequencies of stick transfers in orangutans (and chimpanzees) cannot be explained by greater frequency of their requesting behaviours.

## Discussion

In this study, we aimed to investigate the motivation underlying prosocial behaviour in great apes. More specifically, we examined whether great apes show concern for others as evident in an increased frequency of stick transfers to a conspecific who was harmed compared to a conspecific who was not harmed. Unlike previous studies, this study systematically compared four species of great apes to obtain a more comprehensive picture of their prosocial behaviour.

The results show that overall great apes did not help more after a conspecific was harmed than after a conspecific was not harmed. Only orangutans in the sanctuary transferred sticks after the victim was harmed but in no other condition indicating that they experienced concern for others and thus helped another conspecific. Orangutans at the zoo also frequently helped, but regardless of whether the conspecific was harmed or not. Thus, with the exception of orangutans in a sanctuary, the current study suggests that concern for others may not mediate great apes’ prosocial behaviour.

This finding contradicts results from other mostly observational studies. For example, de Waal [23] emphasizes that there is ample evidence for concern for others in chimpanzees as observed in consolation, in which an uninvolved individual comforts one of the combatants after an aggressive interaction, e.g., by gently putting the arm over the other’s shoulder [45]. However, others argue that feeling with or for others is not essential to display consolation. An alternative explanation is that chimpanzees might comfort others to reduce their own distress in such situations rather than out of genuine sympathy for others [37], [46].

Nevertheless, it is important to note that despite the finding of the current study demonstrating that the majority of great apes did not differentiate between conditions, it is too early to conclude that great apes generally lack the ability to sympathize with and experience concern for others. First, although they might feel concern for others, this may not translate into an action such as helping the harmed individual. The current study, however, did not provide the opportunity for potential other, more direct forms of interaction between the two individuals, such as grooming. Second, stealing food might not be sufficient to cause distress in great apes, although our findings show that particularly the African great apes displayed more arousal behaviours in the Take compared to the Give condition while interacting with the experimenter. It could be that the observed levels of arousal were not sufficient to elicit a response of the helper; however, due to ethical issues, we did not want to use situations that might elicit more intense distress. Third, in contrast to other studies investigating helping behaviour of great apes [6], [34], [35], [47], we tested each dyad only once in each condition, because we were interested in spontaneous helping behavior and predicted that if the effect exists, it should emerge in the first trial right after they have seen someone being harmed. Finally, this study found that at least orangutans in the sanctuary differentiated between conditions. We still treat this result with caution because of the limited sample size and because there are different explanations for the differences between the two populations. For example, as opposed to the orangutans at the zoo, the orangutans at the sanctuary belonged to a different subspecies (Bornean orangutans), were mostly subadults, and were mostly raised by humans. Thus, there are several confounding factors that might account for the differences between the two populations of orangutans and more research is needed to investigate this in more detail.

Orangutans as a group also differed from the other species in the degree of instrumental helping regardless of whether harm occurred or not. Thus, they transferred sticks more when they were needed (in the prosocial situations of the Take and Give conditions) than when they were not needed (in the two control conditions), while chimpanzees did not differentiate between these conditions. Furthermore, orangutans mostly actively offered the sticks to the victims, while the few stick transfers in chimpanzees were mostly tolerated takings and thus passive transfers. In summary, we found no evidence for prosocial behaviours in chimpanzees. While this is consistent with some studies [30]–[32], other studies have reported instrumental helping in this species [5], [6], [34], [47]. This is surprising, since our study included several aspects that have been shown to promote helping in chimpanzees: First, similar to the studies by Warneken and colleagues [6], the current study focused on interactions between conspecifics and involved helping rather than the sharing of food, since several studies have demonstrated that interactions involving food reduce the likelihood of prosocial behaviours ([30], , but see [48]). Second, since Warneken and Tomasello [5] suggested that chimpanzees might not help in cognitively more demanding situations, perhaps because they are not capable of inferring the other’s needs, we conducted warm-up trials with both the victim and the helper on each testing day to make sure the apes understood the characteristics of the situation and knew a tool is needed in the prosocial phase to obtain the food. Furthermore, Yamamoto and colleagues [47] demonstrated that chimpanzees are indeed capable of recognizing others’ needs and consequently transfer the appropriate tool to a conspecific that is confronted with different tool use situations. Finally, a possible explanation for the absence of helping behaviour in chimpanzees in the current study could be that the prosocial phase (3 minutes) was too short for the helper to realize that the victim needed the stick. However, other studies have demonstrated that even shorter durations are sufficient for chimpanzees to help a conspecific [6], [48].

We suggest that a more likely explanation for the absence of helping behaviour in chimpanzees in our study lies in the composition of dyads. In contrast to other studies, we combined the apes with multiple partners with no possibility for repeated interactions and no possibility for reciprocation. This is different from other studies with chimpanzees where individuals repeatedly interacted with the same partner and then changed their roles within the same dyad [35], or where few specific individuals were chosen as recipients of prosocial behaviour, resulting in both recipients and helpers keeping their roles throughout the study [6], [48]. Helping behaviour in chimpanzees might emerge only in these very specific situations regarding the social relationship and the recent interaction history of individuals [29].

It is important to note, however, that despite these methodological differences between the current and previous studies that might explain the inconsistent results regarding the helping behaviour of chimpanzees, we found evidence that orangutans, in contrast to the other apes, transferred sticks in those conditions in which a tool was needed. Therefore it seems unlikely that this task was in general cognitively too demanding or not appropriate to elicit helping behaviours. One further possible explanation for the current differences between species in the propensity to help others is that orangutans are more motivated to exchange objects with others, which is supported by a study that compared the exchange of tokens in four species of great apes [49]. It revealed that orangutans were distinct from the other species, since they consistently exchanged tokens and because most of their interactions were not passive transfers but active offers, similar to the findings of the current study. Thus, orangutans exchange objects more readily than other species, which might increase the likelihood to help by offering objects. Why this is the case for orangutans but not the other great apes, however, remains an open question.

In regard to a possible influence of the victims’ requesting behaviours on the helping behaviour of the helper, there are studies that show that orangutans gestured more than other species to request tokens from their partner [49] and that chimpanzees are more likely to help after their partner performed a request, while spontaneous helping occurred only rarely [35]. However, in the current study, orangutans did not differ from the other species in regard to their frequency of requests, and across species the overall frequency of requests did not predict the likelihood of stick transfers. In other words, we did not find evidence that helping was merely driven by the victim’s requests.

While the sample size for gorillas in the current study was very small, which might at least partly explain the absence of helping in this species, it remains unclear why there were virtually no instances of stick transfers in bonobos. Interestingly, the single instance of a stick transfer in bonobos occurred in a mother-infant dyad indicating that acts of prosocial behaviour are most likely to be directed toward kin [23]. The current study, however, did not specifically address this question, partly because of the nature of the statistical analysis. Since individuals were tested in dyads, the analysis needed to control for a potential influence of the victim’s and helper’s identity on the frequency of stick transfers. As a consequence, despite the considerable number of individuals and dyads, we were not able to consider kin as an additional factor. However, the observed instances of helping in orangutans and chimpanzees are most likely not explained by kin relationships, since the individuals in the two sanctuaries were not related to each other but still transferred sticks. This is supported by a study on chimpanzees that found no evidence that instrumental helping in chimpanzees occurs particularly often in mother-infant dyads [33].

In summary, this study showed that prosocial behaviours in great apes is most likely not motivated by the ability to feel concern for others, although there was some evidence that orangutans help more after witnessing others being harmed. This differentiates nonhuman great apes from human infants, who – in a similar experimental setting - helped more after they observed another human being harmed, even in the absence of any behaviours indicating this person’s distress [20]. Furthermore, the lack of helping in chimpanzees – as opposed to orangutans - contrasts with positive evidence from several other studies. These inconsistent findings demonstrate that prosocial behaviour in chimpanzees and most likely other primate species depends very much on the design of the study and thus might be influenced by many variables, such as the identity and relationship of the interacting individuals, or whether food is involved or not [28], [29], [33]. Furthermore, although great apes might not help immediately after observing another conspecific being harmed, they might behave differently in their later interactions with that individual. Therefore, future research needs to address the subsequent and more direct interactions between individuals to further our understanding of what motivates prosocial behaviour in nonhuman primates.

## Supporting Information

Table S1 **Tested individuals.** Individuals of the four species that were part of any dyad (as victim and/or helper) together with their location and information on their individual characteristics.
(DOCX)

Table S2 **Tested dyads.** They consist of the victim (first name) and the helper, shown for each species and population.
(DOCX)

Video S1 **Stick transfer after request in the prosocial phase of the Take condition in orangutans (victim: Dokana, helper: Padana).** Helper approaches mesh (Camera 03: 09:50:53); victim approaches the mesh (Camera 02: 09:50:57); victim performs a quick ‘hand shake’ (Camera 02: 09:50:58); then helper offers stick (Camera 02: 09:50:59); victim obtains food (Camera 01: 09:52:23). (Camera 01: Victim, Camera 02: Interactions between victim and helper, Camera 03: Helper).
(DOCX)

Video S2 **Stick transfer without request in the prosocial phase of the Take condition in orangutans (victim: Kila, helper: Padana).** Helper approaches and puts stick through mesh (Camera 11:28:17); victim approaches mesh and takes offered stick (Camera 2: 11:28:34); victim obtains the food (Camera 1: 11:28:34). (Camera 01: Victim, Camera 02: Interactions between victim and helper, Camera 03: Helper).
(DOCX)

Video S3 **Stick transfer in the prosocial phase of the Take condition in gorillas (victim: Louna, helper: Kibara).** Helper (right) puts sticks through mesh (43:46), victim approaches (from the left) and takes sticks (43:52).
(DOCX)
