# Correction: Does Sympathy Motivate Prosocial Behaviour in Great Apes?

**DOI:** 10.1371/annotation/1fe9c2b8-84dd-44c4-a4ba-b62e0460b513

**Published:** 2014-01-29

**Authors:** Katja Liebal, Amrisha Vaish, Daniel Haun, Michael Tomasello

The files extensions for the supporting video files were erroneously changed making them difficult to open. The video titles and legends on the article page are still correct. Please download the correct videos here:

Video S1, Video S2, Video S3: http://www.plosone.org/corrections/video_s1_s2_s3.zip

