# Supplementary material for: Does Sympathy Motivate Prosocial Behaviour in Great Apes?
Source: PLoS One. 2014 Jan 8;9(1):e84299. doi: 10.1371/journal.pone.0084299 (PMC3885567; doi:10.1371/journal.pone.0084299)
Supplement: Table S1 — Tested individuals. Individuals of the four species that were part of any dyad (as victim and/or helper) together with their location and information on their individual characteristics. (DOCX) [file pone.0084299.s001.docx]

**Table S1. Tested individuals.** Individuals of the four species that were part of any dyad (as victim and/or helper) together with their location and information on their individual characteristics.

| **Species** | **Location** | **Name** | **Age (in years)** | **Sex** |
| --- | --- | --- | --- | --- |
| Orangutans | WKPRC | Raja* | 6 | F |
|  |  | Kila* | 9 | F |
|  |  | Padana* | 11 | F |
|  |  | Dokana | 20 | F |
|  |  | Pini* | 21 | F |
|  |  | Bimbo | 29 | M |
|  |  | Dunja | 36 | F |
|  | OCCQ | Galih | 6 | M |
|  |  | Ari | 7 | M |
|  |  | Rowland | 7 | M |
|  |  | Sabin* | 7 | M |
|  |  | Sam* | 7 | M |
|  |  | Diva* | 8 | F |
|  |  | Yasmin* | 8 | F |
|  |  | Cabang | 8.5 | F |
|  |  | Edwin* | 8.5 | M |
|  |  | Kraba | 9 | F |
|  |  | Mercedes | 9 | F |
|  |  | Sallie* | 9.5 | F |
|  |  | Ulin | 11 | F |
|  |  | Bali* | 13 | F |
| Gorillas | WKPRC | Louna* | 4 | F |
|  |  | Kibara* | 6 | F |
|  |  | Viringika* | 15 | F |
|  |  | Gorgo | 29 | M |
| Chimpanzees | WKPRC | Alex* | 9 | M |
|  |  | Annett | 10 | F |
|  |  | Alexandra* | 10 | F |
|  |  | Fifi | 16 | F |
|  |  | Gertruida* | 16 | F |
|  |  | Jahaga | 16 | F |

**Table S1.** continued

| **Species** | **Location** | **Name** | | **Age (in years)** | **Sex** |
| --- | --- | --- | --- | --- | --- |
| Chimpanzees | Ngamba Island | | Okech | 10 | M |
|  |  | | Nani | 10 | F |
|  |  | | Indi | 12 | M |
|  |  | | Yoyo | 12 | F |
|  |  | | Kazahukire | 12 | F |
|  |  | | Pasa | 12 | F |
|  |  | | Namukisa | 12 | F |
|  |  | | Bwambale | 12 | M |
|  |  | | Baluku | 13 | M |
|  |  | | Bili | 13 | F |
|  |  | | Asega | 13 | M |
|  |  | | Umugenzi | 14 | M |
|  |  | | Kalema | 15 | M |
|  |  | | Umutama | 15 | M |
|  |  | | Mawa | 15 | M |
|  |  | | Nkuumwa | 15 | F |
|  |  | | Ikuru | 16 | F |
|  |  | | Sally | 20 | F |
|  |  | | Becky | 20 | F |
|  |  | | Tumbo | 22 | M |
|  |  | | Robbie | 25 | M |
|  |  | | Sophie | 25 | F |
|  |  | | Kidogo | 27 | F |
| Bonobos | WKPRC | | Joey* | 27 | M |
|  |  | | Kuno | 13 | M |
|  |  | | Limbuko* | 14 | M |
|  |  | | Luiza* | 4 | F |
|  |  | | Ulindi | 16 | F |
|  |  | | Yasa* | 12 | F |

* = individuals that first participated as victims; the remaining individuals first participated as helpers; WKPRC = Wolfgang Köhler Primate Research Center, Leipzig, Germany; OCCQ = Orangutan Care Center and Quarantine Pasir Panjang, Kalimantan, Indonesia
