# Supplementary material for: Does Sympathy Motivate Prosocial Behaviour in Great Apes?
Source: PLoS One. 2014 Jan 8;9(1):e84299. doi: 10.1371/journal.pone.0084299 (PMC3885567; doi:10.1371/journal.pone.0084299)
Supplement: Table S2 — Tested dyads. They consist of the victim (first name) and the helper, shown for each species and population. (DOCX) [file pone.0084299.s002.docx]

**Table S2. Tested dyads.** They consist of the victim (first name) and the helper, shown for each species and population.

| **Species** | **Population** | **Dyad** |
| --- | --- | --- |
| Orangutans | WKPRC | Bimbo - Dokana |
|  |  | Bimbo - Kila |
|  |  | Dokana - Dunja |
|  |  | Dokana - Padana |
|  |  | Dokana - Pini |
|  |  | Dunja - Bimbo |
|  |  | Dunja - Padana |
|  |  | Dunja - Pini |
|  |  | Kila - Dokana |
|  |  | Kila - Dunja |
|  |  | Kila - Padana |
|  |  | Padana - Bimbo |
|  |  | Padana - Pini |
|  |  | Pini - Bimbo |
|  |  | Pini - Kila |
|  |  | Pini - Raja |
|  |  | Raja - Dokana |
|  |  | Raja - Dunja |
|  | OCCQ | Bali - Sabin |
|  |  | Diva - Mercedes |
|  |  | Edwin - Roland |
|  |  | Kraba - Harry |
|  |  | Mercedes - Salih |
|  |  | Roland -Yasmin |
|  |  | Sabin - Mercedes |
|  |  | Salih - Kraba |
|  |  | Sam - Diva |
|  |  | Ulin - Galih |
|  |  | Yasmin - Jabang |
| Gorillas | WKPRC | Gorgo - Viringika |
|  |  | Kibara - Gorgo |
|  |  | Louna - Kibara |
|  |  | Louna - Viringika |
|  |  | Viringika - Kibara |
| Bonobos | WKPRC | Joey - Kuno |
|  |  | Joey - Limbuko |
|  |  | Joey - Yasa |
|  |  | Limbuko - Kuno |
|  |  | Luisa - Joey |
|  |  | Luisa - Ulindi |
|  |  | Ulindi - Joey |
|  |  | Ulindi - Kuno |
|  |  | Ulindi - Limbuko |
|  |  | Yasa - Kuno |
|  |  | Yasa - Luisa |
|  |  | Yasa - Ulindi |

**Table S2** continued**.**

| **Species** | **Population** | **Dyad** |
| --- | --- | --- |
| Chimpanzees | WKPRC | Alex - Alexandra |
|  |  | Alex - Annett |
|  |  | Alex - Getruida |
|  |  | Alexandra - Fifi |
|  |  | Alexandra - Jahaga |
|  |  | Annett - Alexandra |
|  |  | Annett - Gertruida |
|  |  | Fifi - Alex |
|  |  | Fifi - Annett |
|  |  | Fifi - Gertruida |
|  |  | Gertruida - Alexandra |
|  |  | Gertruida - Jahaga |
|  |  | Jahaga - Alex |
|  |  | Jahaga - Annett |
|  |  | Jahaga - Fifi |
|  | Ngamba Island | Bili - Asega |
|  |  | Indi - Ikuru |
|  |  | Kalema - Umutama |
|  |  | Kazahukire - Yoyo |
|  |  | Kidogo - Mawa |
|  |  | Mawa - Pasa |
|  |  | Namukisa - Sophie |
|  |  | Nkuumwa - Bwambale |
|  |  | Okech - Robbie |
|  |  | Sally - Becky |
|  |  | Tumbo - Baluku |
|  |  | Umugenzi - Nani |

WKPRC = Wolfgang Köhler Primate Research Center, Leipzig, Germany; OCCQ = Orangutan Care Center and Quarantine Pasir Panjang, Kalimantan, Indonesia
